# Measurement and Modeling of Narrowband Channels for Ultrasonic Underwater Communications

**DOI:** 10.3390/s16020256

**Published:** 2016-02-19

**Authors:** Francisco J. Cañete, Jesús López-Fernández, Celia García-Corrales, Antonio Sánchez, Encarnación Robles, Francisco J. Rodrigo, José F. Paris

**Affiliations:** 1 Departamento de Ingeniería de Comunicaciones, ETS Ingeniería de Telecomunicación, Universidad de Málaga, Málaga 29071, Spain; jlf@ic.uma.es (J.L.-F.); celia@ic.uma.es (C.G.-C.); paris@ic.uma.es (J.F.P.); 2 Sociedad Anónima de Electrónica Submarina (SAES), Cartagena 30205, Spain; a.sanchez@electronica-submarina.com (A.S.); e.robles@electronica-submarina.com (E.R.); f.rodrigo@electronica-submarina.com (F.J.R.)

**Keywords:** sensor networks, underwater acoustic communications (UAC), fading channels, statistical channel modeling, narrowband channel measurements, Doppler spread, parameter estimation, channel ergodic capacity, *κ*-*μ* shadowed, Rice shadowed

## Abstract

Underwater acoustic sensor networks are a promising technology that allow real-time data collection in seas and oceans for a wide variety of applications. Smaller size and weight sensors can be achieved with working frequencies shifted from audio to the ultrasonic band. At these frequencies, the fading phenomena has a significant presence in the channel behavior, and the design of a reliable communication link between the network sensors will require a precise characterization of it. Fading in underwater channels has been previously measured and modeled in the audio band. However, there have been few attempts to study it at ultrasonic frequencies. In this paper, a campaign of measurements of ultrasonic underwater acoustic channels in Mediterranean shallow waters conducted by the authors is presented. These measurements are used to determine the parameters of the so-called *κ*-*μ* shadowed distribution, a fading model with a direct connection to the underlying physical mechanisms. The model is then used to evaluate the capacity of the measured channels with a closed-form expression.

## 1. Introduction

Underwater sensor nodes and networks allow a new way to gather data in real time from the submarine environment for applications like pollution monitoring, supervision of undersea equipment of oil and gas companies, search and survey missions, seismic prediction, fish farming maintenance, *etc*. The underwater medium is known to be one of the most challenging communication channels currently in use today. Three technologies have been considered for this purpose over the years: acoustic, optical and electromagnetic. The severe drawbacks of the optical and electromagnetic alternatives (particularly an extremely high attenuation that limits the transmission range) have made acoustic waves the preferred option in most applications [1,2]. However, the use of acoustic technology is not free of problems. The propagation of an acoustic wave is strongly affected by multiple physical processes whose relative influence is remarkably different from one scenario to another [3]. Basically, the acoustic signal experiences high attenuation and distortion, the latter due to both the multipath propagation and time-variation of the channel. Such time variation has its origin in the movement of the underwater environment, and it causes the received signal level to randomly fluctuate with time, which is referred to as fading.

As will be explained in more detail in the next section, multipath propagation causes frequency selectivity that can be evaluated by estimating the frequency response of the channel or by means of average parameters, like the delay spread or the coherence bandwidth. Regarding the time variation of the channel, it can also be quantified by average parameters, like the coherence time or the Doppler spread, but for a more accurate evaluation of its effect, the fading statistics must be explored [2,4,5,6,7,8,9].

Most systems designed for underwater acoustic communications (UAC) work in low frequency bands, but currently, there is an increasing concern in going beyond the audio band to take advantage of the smaller wavelength at ultrasonic frequencies. This is of particular interest for sensor networks, because the transmitter and receiver devices can have a more reduced size and weight, and they can operate with a lower energy consumption. The channel has been extensively explored for UAC in the audio band [2,3,4,5,7,8,9,10], but there are few works devoted to the channel analysis at the ultrasonic band [1,3,11]. At these frequencies, communications are more exposed to the effect of fading, and its modeling becomes more important. In this paper, our attention will be centered on the analysis of the channel time variation with the characterization of such fading. We are focusing on telemetry applications that require low digital transmission speeds, and hence, the transmission bandwidth can be considered narrow enough to assume a frequency flat channel (*i.e*., there is no distortion due to frequency selectivity).

The paper is organized as follows. Section 2 presents a review of the state-of-the-art channel modeling for UAC, including fading models. In Section 3, the measurement procedure and results on actual channels are described. In Section 4, the measurements results are fit to the *κ*-*μ* shadowed fading model, and in Section 5, the ergodic capacity of the measured channels is analyzed. Finally, some conclusions are summarized in Section 6.

## 2. Channel Characterization for UAC

**Figure 1:**
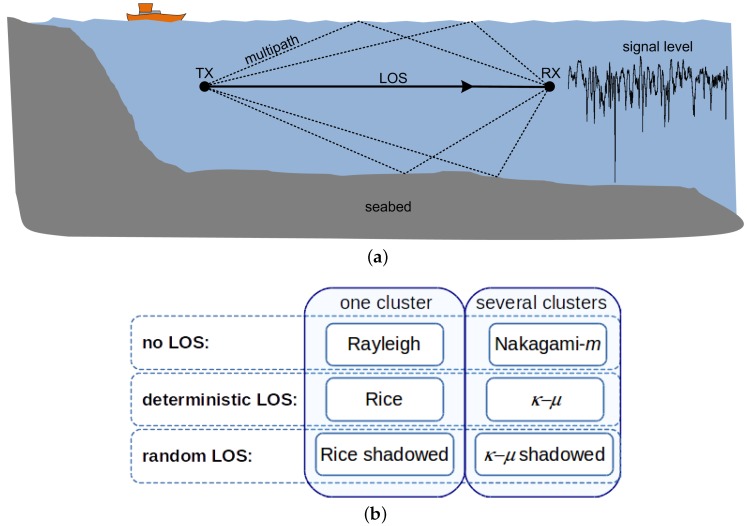
Channel modeling diagrams: (**a**) example of underwater acoustic communications system; (**b**) classification of fading models.

The main factors that adversely affect the propagation of a signal in an UAC channel are:

- *Attenuation*: In UAC channels the attenuation is, in general terms, much higher than in terrestrial radio frequency (RF) channels and strongly depends on frequency and distance. Empirical formulas to predict the *path loss*, or mean attenuation, are available in the literature [12]. The mechanisms responsible for the path loss are basically signal *absorption* (part of the signal energy is transferred to the medium) and signal *spreading* (which depends on the geometry of propagation). Absorption increases rapidly with frequency, while spreading does with distance, yielding a limited range and limited bandwidth channel.
- *Distortion*: This is due to *multipath propagation* and *fading*. Multipath propagation in shallow UAC is caused by the reflections of the acoustic signal on the seabed, the surface of the sea and any other objects, and the different paths undergo different delays at reception that result in a dispersion in time (it can also be seen as the superposition of constructive and destructive contributions selective in frequency) [3]. A common way to characterize the time dispersion of the channel, or its frequency selectivity, is by evaluating parameters like the *coherence bandwidth* or the *delay spread*, which is several orders of magnitude higher than those measured in RF channels [13] due to the relatively low speed of sound in water. A typical propagation scenario is shown in Figure 1a, where a main component (line of sight (LOS)) is represented along with various multipath components. This multipath scenario is not static, but varies with time due to several factors, like surface waves (that change the reflection angles of the multipath components), internal waves, the motion of transmitter/receiver, changes in the medium physical conditions, *etc*. The time variation results in a fluctuation of the received signal level, the so-called fading. This fluctuation may also be experienced by the LOS component, a phenomenon referred to as *shadowing*. A simple way to characterize the time variation is directly, through the *coherence time*, or indirectly, through the *Doppler spread*, which measures the frequency broadening. This effect can be remarkably intense in UAC due, again, to the relatively low speed of sound in water.

One step further in the evaluation of the distortion caused by the time variation of the channels is the statistical modeling of the fading. Different models correspond to different assumptions on the ray tracing from transmitter to receiver. Ray tracing can be considered a valid approximation in ultrasonic UAC, since the involved wavelengths are in the range of cm, similar to microwaves in electromagnetic communications. Three main assumptions are taken into consideration for modeling the fading: (1) the existence of a LOS component or not; (2) the deterministic or random behavior of the LOS component; and (3) the possibility of the signal traveling through different clusters of multipath waves. In the next section, a review of different fading models is presented.

### UAC Fading Models Review

There is no general agreement about the best suited distribution model for the statistical characterization of this kind of channel. Several statistical fading models have been proposed in the literature. Although the main attribute of a model is its ability to fit experimental data, other features must be taken into account, for instance the easiness for mathematical manipulation and the existence of an underlying physical mechanism that justifies the model. Here are the main distribution models addressed for underwater acoustic channels, most of them also employed in terrestrial RF scenarios [13,14,15]:

- *Rayleigh distribution*: This corresponds to a situation where a high number of multipath fluctuating components arrive at the receiver and there is no direct path, *i.e*., no LOS component [1,7,10].
- *Rice distribution*: This is a generalization of the Rayleigh distribution used when a LOS component with non-fluctuating level is added to the weaker multipath fluctuating components of the previous scenario [6,7,8,9].
- *Nakagami-m distribution*: This is another generalization of the Rayleigh distribution, but considering the case where *m* clusters of multipath waves propagate in a non-homogeneous environment and arrive at the receiver with no main components within each cluster (strictly speaking, only for a cluster without reflections, the main component represents a LOS path; for the remaining clusters, with some reflections, the main component corresponds to the ray tracing for the reflected path). The envelope of each cluster signal is hence considered Rayleigh distributed. Nakagami-*m* with m=1 is Rayleigh [16].
- *κ-μ distribution*: This consists of a generalization of the Nakagami-*m* distribution where a non-fluctuating main component is considered within each cluster [17]. Actually, the *κ*-*μ* distribution is a generalization of the three previous distributions. Nakagami-*m* is approached if the level of the LOS components tends to zero. Rice is obtained if the number of clusters is set to one, and Rayleigh is obtained if both particularizations are simultaneously assumed.
- *K distribution*: This is a mixture of Gamma and Rayleigh distributions. The underlying mechanism for signal fluctuation that leads to this distribution is not clear (a question may arise about if some of the models can be regarded (entirely or partially) as merely fitting functions) [18].
- *Rice shadowed distribution*: This represents a generalization of the Rice distribution where the main component is assumed to be a random Nakagami-*m* variable [19]. It models the case where the LOS component experiences shadowing.
- *κ-μ shadowed distribution*: This is a generalization of the *κ*-*μ* distribution where the main components are considered Nakagami-*m* random variables. This distribution includes Rayleigh, Rice, Rice shadowed, Nakagami-*m* and *κ*-*μ* as particular cases. A better behavior of the *κ*-*μ* shadowed model over the rest has been addressed in [20] for UAC.

A summary of these models is shown in Figure 1b (where the *K* distribution is intentionally not included).

## 3. Ultrasonic Measurements for UAC

In this section, a measurement campaign to characterize ultrasonic UAC channels is addressed. The measurement procedure is briefly described, and some of the obtained results are discussed.

### 3.1. Channel Measurements Procedure

The results presented here are part of a measurement campaign in the framework of the underwater communication experiments (UCEX), which is a multi-year measurement project of the company SAES and the Universidad de Málaga. This campaign was carried out in the Mediterranean Sea in La Algameca Chica (Cartagena, Spain), in shallow waters with a sandy seabed (and depths from 14 to 30 m approximately). The measurements presented here were registered during the same day in November 2013 and with smooth sea conditions (World Meteorological Organization sea state Code 2, waves of less than 0.5 m). The different link distances tested between the transmitter and the receiver were 50, 100 and 200 m; and the projector and the hydrophone were suspended from anchored boats with different cable lengths to reach a depth of 3, 6 and 9 m.

**Figure 2:**
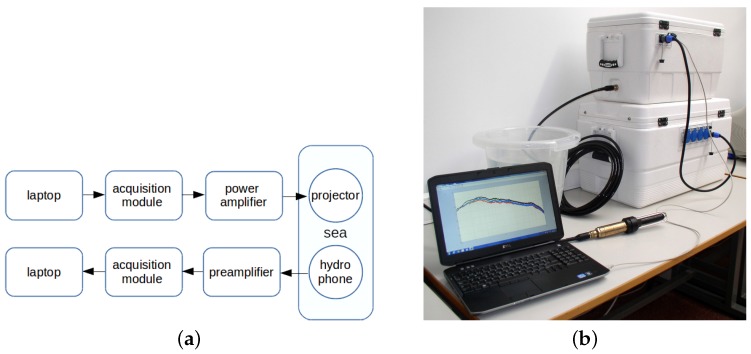
Measurements setup: (**a**) block diagram of the measurement equipment; (**b**) picture of the receiver part at the laboratory.

The measurement setup used for the UCEX is sketched in Figure 2, where a picture of the equipment employed at the receiver is also shown. The setup comprises: two laptops (for signals control and storage and off-line signal processing for monitoring purposes); two acquisition modules, IOtech Personal DAQ3000 (with 16 bits of resolution and a 1-MHz sample rate, which were used as a digital-to-analog converter at the transmitter and as an analog-to-digital converter at the receiver); Brüel & Kjaer 2713 power amplifier and Brüel & Kjaer hydrophone 8105 at the transmitter; Reson VP2000 voltage preamplifier EC6081 (with a 1-MHz bandwidth) and Reson TC4032 low noise hydrophone at the receiver.

The equipment allows performing accurate noise and channel measurements in the ultrasonic band. The sounding signals were sinusoids of constant amplitudes and frequencies of 32, 64 and 128 kHz. The received signals were recorded during a time window of 60 s with a 1-MHz sample rate. Afterwards, the records were digitally post-processed, applying first a 400-Hz bandwidth bandpass filter centered at the frequency of the transmitted sinusoid. The function of the bandpass filter was to attenuate the out of band interference and noise. Its bandwidth was set to a value larger than the expected Doppler spread. This way, the spectral spreading caused by the channel on the sounding signal was not distorted.

### 3.2. Measurement Results

**Figure 3:**
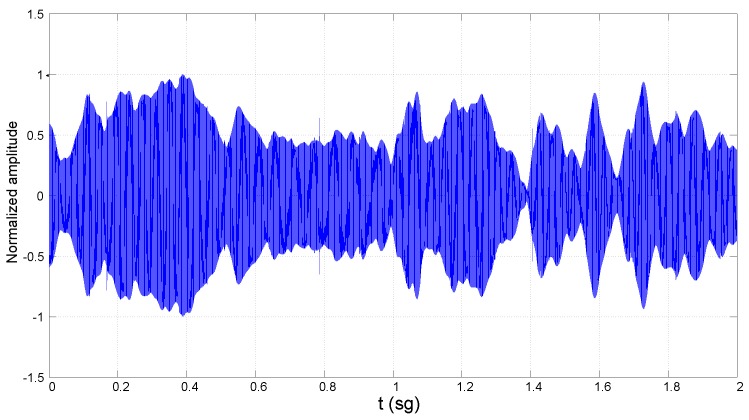
Segment of the received signal in Channel A6-128.

The set of measured channel configurations is described in Table 1, including the sounding signal frequency (SF), the transducers’ depth (TD), the transducers’ separation (TS) or link distance and the average sea depth (ASD). A segment of the captured signal on reception on Channel A6-128 is shown in Figure 3 as an example where the signal level fluctuation due to fading is evident. From this type of signal, several parameters of the channel behavior can be extracted (prior to do that, the stationarity of the channels has been confirmed by comparing the CDF of successive measurements in the same scenario).

**Figure 4:**
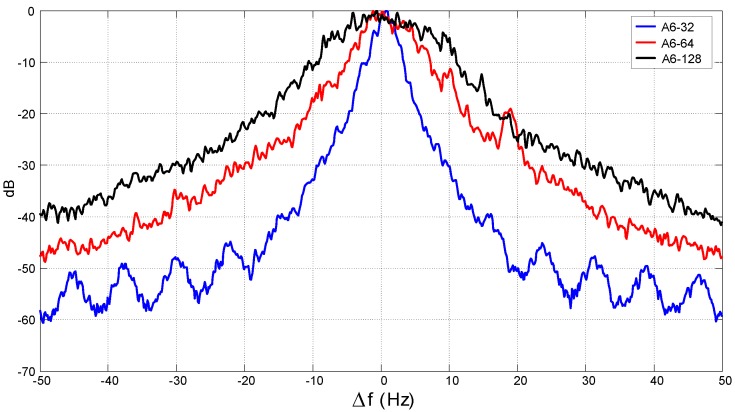
Normalized Doppler spectrum in Channels A6-32, A6-64 and A6-128.

- *Channel attenuation*. This parameter is an estimation, using short time averaging, of the instantaneous attenuation (the inverse of the channel gain) measured from the registered data (not to be confused with path loss). The change in the channel gain produced by the fading is evaluated from the measurements. The range of values observed in the channel attenuation is shown in Table 2 for each group of measurements: *i.e*., channels with the A code (TS = 50 m), B code (TS = 100 m) and C code (TS = 200 m).
- *Coherence time* / *Doppler spread*: The small time scale variation of the underwater environment produces a variant shift of the received signal frequency due to the Doppler effect. A way of quantizing the variation of the channel is through the Doppler spread, defined as the rms (root mean square) bandwidth [13] of the received signal spectrum when a pure tone is transmitted (the so-called Doppler spectrum). An alternative parameter is the coherence time, which is inversely proportional to the Doppler spread. It measures the period over which the channel can be considered time invariant. Different criteria are considered in the literature to determine the inverse proportionality constant between both parameters. The one selected here yields the relation coherence time = 0.2/Doppler spread [13]. In Figure 4, the Doppler spectra measured in Channels A6-32, A6-64 and A6-128 are shown. The values obtained for the coherence time and Doppler spread for each channel are listed in Table 2.
- Fading statistics: Fading has been characterized by measuring the fluctuations on the channel power gain, $\alpha^{2}$, from the fluctuations of the received signal envelope (see Figure 3). The samples of the channel gain *α* are assumed random, stationary and ergodic. The amount of fading (AoF) is defined as the dispersion in the distribution of $\alpha^{2}$, *i.e*., the variance divided by the mean square [15]. A normalization of $\alpha^{2}$ is carried out before data processing, so that $E[\alpha^{2}]=1$ and, hence, AoF can be directly related to the variance. The collected channel gain samples are afterwards processed to obtain the cumulative distribution function (CDF) of the channel power gain.
  The procedure to estimate the fading does not avoid some in-band noise; however, noise was also registered alone at the measurement location, and the signal to noise ratio obtained at the output of the signal processing algorithms was larger than 30 dB in the worst conditions (for a center frequency of 128 kHz and link distance of 200 m); these results allow one to disregard the noise influence on the estimated fading CDF.

**Figure 5:**
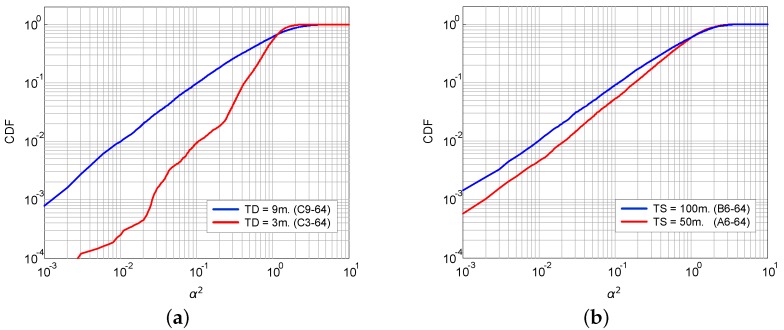
Cumulative distribution function (CDF) of the power gain estimated from measurements: (**a**) for two different transducers’ depth and the same link distance (200 m) and sounding frequency (64 kHz); (**b**) for two different link distances and the same transducers’ depth (6 m) and sounding frequency (64 kHz).

The estimated CDF of the fading of some of the measured channels is shown in Figure 5, for illustrative purposes only. As stated before, the AoF is related to the variance of the distribution that, in turn, determines the steepness of the slope of the corresponding CDF. This means that the higher the steepness, the lower the AoF. In general, it is observed that the CDF shape exhibits a stronger frequency dependence in shorter links [21]. As seen in Figure 5a, the transducers’ depth seems to have influence also on the fading statistics, because the curve for the transducers at less depth shows less dispersion in the distribution (it has a steeper slope). In Figure 5b, the CDF of two channels of a 50-m and a 100-m link distance is shown where the sounding signal frequency is 64 kHz in both measurements. As is observed, in this case, the longer channel exhibits more fading than the shorter one. This is not necessarily the general case, as the fading level depends not only on the link distance, but also on the environment and the deployment geometry.

In the next section, these measurements are used to estimate the channel parameters according to the *κ*-*μ* shadowed model.

## 4. Ultrasonic Fading Modeling for UAC

Among the statistical models considered for the fading shown in Figure 1, the *κ*-*μ* shadowed distribution outperforms others in our measurements of ultrasonic UAC. In the next subsections, details of the model and a verification of the fit to the collected data are presented.

### 4.1. κ-μ Shadowed Distribution

The suitability of the *κ*-*μ* shadowed distribution to explain signal fading in UAC was first studied in [20]. It has an underlying physical model, provides a unification of popular fading models and has good analytical properties (closed forms for the probability density function and other relevant functions are available). The main parameters that define this distribution are:

- *m*-parameter: All of the main components exhibit a common shadowing fluctuation that is represented by a Nakagami-*m* random variable with shaping parameter *m*.
- *μ*-parameter: This represents the number of clusters. Although the number of clusters should be a natural positive number, the parameter *μ* is allowed to take any non-negative real value, which leads to a more general and flexible distribution.
- *κ*-parameter: This represents the ratio between the total power of the main components and the total power of the scattered waves. The main components are also called dominant components, but in the case of *κ* less than one, the term dominant may be confusing.

An empirical validation of this model was conducted in [20], where data from the TREX04 experiment were used to fit the model (that experiment was conducted by the Naval Research Laboratory, in the coast of New Jersey in April 2004, see details in [5]). The measurements were taken in a frequency range below 20 kHz. In the next section, the set of new measurements conducted by the authors is used to confirm the suitability of the *κ*-*μ* shadowed distribution to model the fading also in ultrasonic UAC.

### 4.2. Model Parameters Estimation

The parameters of the *κ*-*μ* shadowed distribution obtained by optimization for the different measured channels are presented in Table 3 (the Nelder–Mead numerical method, implemented in MATLAB routines, has been employed to calculate them). The error factor *ϵ* used to quantify the goodness of fit between the CDF $F_{\alpha^{2}}\left(\cdot \right)$ of the fading estimated from the measurements and the analytical CDF ${\hat{F}}_{\alpha^{2}}\left(\cdot \right)$ calculated from the model with those parameters is given by:

(1) $$\epsilon =\max_{x}\left|\log_{10}{\hat{F}}_{\alpha^{2}}\left(x;\kappa ,\mu ,m\right)-\log_{10}F_{\alpha^{2}}\left(x\right)\right|$$

with:

(2) $$\begin{array}{c} {\hat{F}}_{\alpha^{2}}\left(x;\kappa ,\mu ,m\right)=\frac{\mu^{\mu -1}m^{m}{\left(1+\kappa \right)}^{\mu }}{\Gamma \left(\mu \right){\left(\mu \kappa +m\right)}^{m}}x^{\mu }\\ \;\times \Phi_{2}\left(\mu -m,m;\mu +1;-\mu \left(1+\kappa \right)x,-\mu \left(1+\kappa \right)\frac{mx}{\mu \kappa +m}\right) \end{array}$$

where Γ(·) is the Gamma function and $\Phi_{2}$ is the bivariate confluent hypergeometric function [22]. This error factor is a modified version of the well-known Kolmogorov-Smirnov (KS) statistic [23], but the logarithm of the CDF is employed instead of the CDF itself, to outweigh the fit in values close to zero (when fading is more severe). This region is of special interest in communication systems, since it determines some performance measures, like the outage probability or the bit error rate.

**Figure 6:**
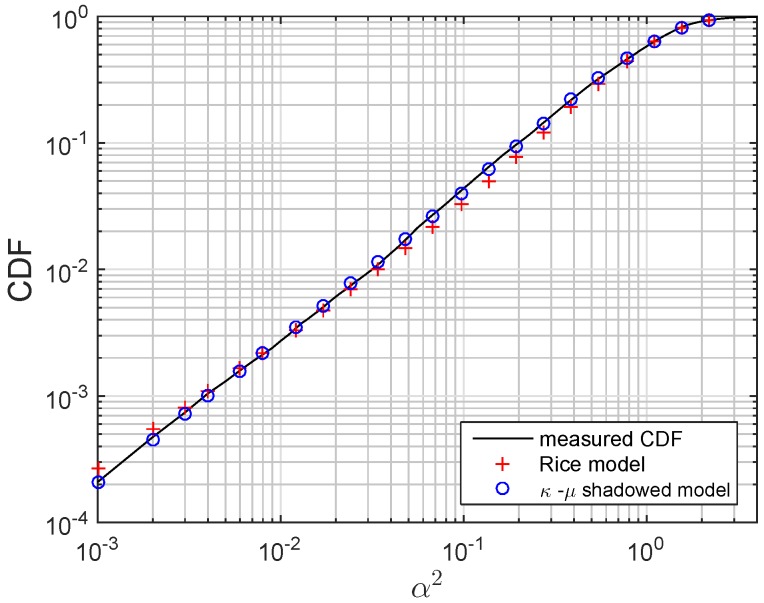
Example of the κ-μ model fit and the Rice model fit for one of the measured channels: C9-32.

An example of the model fit is presented in Figure 6, where the experimental CDF of the power gain measured in one of the channels is plotted *vs*. the analytical CDF of the modeled channel. The latter curve is the result of using the set of parameters in Table 3 and calculating the CDF according to Equation (2). The CDF obtained using the Rice model, the most common in UAC, is also shown in Figure 6. (For the Rice distribution, the so-called fading parameter *K* is used, which represent the ratio of the power in the LOS component to the power in the other multipath components [14].) The error factor obtained for the fit of this channel is 0.111, while for the *κ*-*μ* shadowed, it is 0.026, considerably lower. As seen in Table 3, the fit of all of the measured channels with the two models exhibits a similar behavior, which supports the conclusion that the *κ*-*μ* shadowed distribution is better to model underwater channels also in the ultrasonic band.

## 5. Analysis of the Ultrasonic Channels’ Capacity

The ergodic capacity (in bit/s/Hz, [14]) for a channel modeled with the *κ*-*μ* shadowed distribution can be calculated using the exact closed-form expression presented in [24]. For this evaluation, the following assumptions apply: The received noise is approximated as Gaussian (although the presence of some non-Gaussian components has been reported [4]), and the noise power spectral density is constant in the bandwidth of interest (this was verified in the measurements), so that the statistics of the received signal to noise ratio *γ* are determined by the statistics of the signal received power, $\alpha^{2}$, *i.e*., the fading statistics. Under these circumstances, the ergodic capacity C is given by [14].

**Figure 7:**
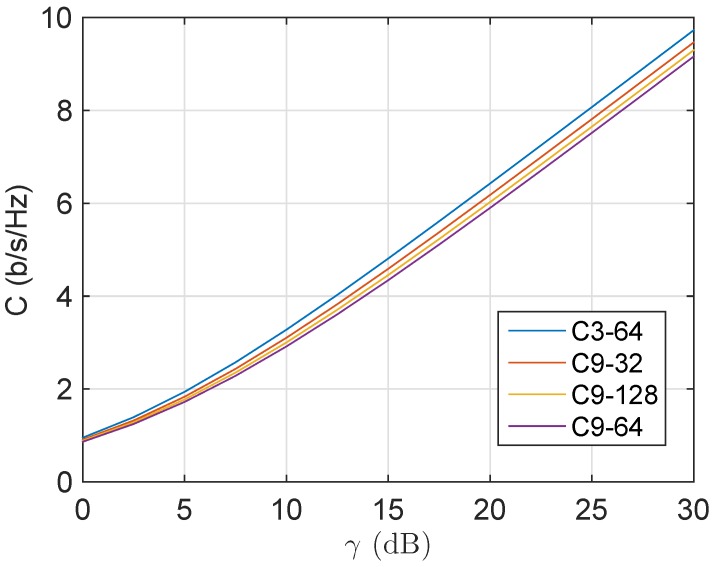
Evaluation of the ergodic capacity (by means of the model) for the measured channels with TS = 200 m and ASD = 25 m.

In Figure 7, the ergodic capacity of several measured channels is depicted as a function of the average signal to noise ratio. The capacity column in Table 3 represents the ergodic capacity of each channel for a received average signal to noise ratio of 12.5 dB by using the expressions in [24]. The capacity obtained from the model agrees with the physical interpretation that the higher the fading, the lower the capacity. Knowing the magnitude order of the capacity of these underwater channels is crucial for the design of a communication link. In the channels under test, it is around 4 b/s/Hz for a 12.5-dB signal to noise ratio, which means that a 1-kHz bandwidth system could theoretically transmit around 4 kbps free of errors.

(3) $$C=\int_{0}^{\infty }log_{2}\left(1+\gamma \right)f_{\gamma }\left(\gamma \right)d\gamma$$

where $f_{\gamma }\left(\gamma \right)$ is the probability density function (PDF) associated with the fading CDF in Equation (2).

## 6. Conclusions

In this paper, the channel in ultrasonic UAC is studied, an essential point to foster the development of more efficient ultrasonic underwater modems that can be employed in sensor nodes and sensor networks. A campaign of measurements in Mediterranean shallow waters is presented and analyzed to extract parameters like the channel attenuation, Doppler spread and the fading statistics. The problem of modeling the fading in such channels is also addressed, and a review of different classical statistical models is provided, with emphasis on a model that has been recently proposed for this environment: the *κ*-*μ* shadowed distribution. The measurement results are used to validate the usefulness of this model in the ultrasonic band. Moreover, a closed-form expression obtained from the model has been used to calculate the ergodic capacity of the channels.

## Figures and Tables

**Table 1:** Summary of measured channel characteristics. SF: Sounding signal frequency, TD: Transducers’ depth; TS: Transducers’ separation; ASD: Average sea depth.

Channel Code	SF (kHz)	TD (m)	TS (m)	ASD (m)
A6-32	32	6	50	16
A6-64	64	6	50	16
A6-128	128	6	50	16
B6-32	32	6	100	20
B6-64	64	6	100	20
B6-128	128	6	100	20
C3-64	64	3	200	25
C9-32	32	9	200	25
C9-64	64	9	200	25
C9-128	128	9	200	25

**Table 2:** Measured channel attenuation, Doppler spread and coherence time.

Channel Code	Channel Attenuation (dB)	Doppler Spread (Hz)	Coherence Time (ms)
A6-32	30–50	1.7	116.3
A6-64		4.0	50.2
A6-128		6.0	33.5
B6-32	35–65	2.5	78.9
B6-64		4.2	47.4
B6-128		6.7	30.0
C3-64	50–80	2.8	70.5
C9-32		1.7	119.6
C9-64		2.6	76.8
C9-128		4.0	50.2

**Table 3:** Fitting parameters of the κ-μ distribution and the Rice distribution for the measured channels.

	*κ*-*μ* Model					Rice Model	
Channel Code	*κ*	*μ*	*m*	*ϵ*	*C(b/s/Hz)*	*K*	*ϵ*
A6-32	2.33	0.92	1.86	0.029	3.67	0.55	0.068
A6-64	2.90	1.00	3.18	0.061	3.78	1.71	0.061
A6-128	9.56	1.27	1.67	0.114	3.81	3.44	0.23
B6-32	3.03	0.91	2.15	0.056	3.71	0.21	0.085
B6-64	1.89	0.94	1.32	0.049	3.61	0.00	0.159
B6-128	1.99	1.01	1.86	0.022	3.69	1.01	0.028
C3-64	7.66	0.90	18.68	0.192	4.03	5.67	0.197
C9-32	4.06	1.13	2.45	0.026	3.83	2.64	0.111
C9-64	0.03	1.02	6.32	0.053	3.61	0.03	0.104
C9-128	1.56	1.04	2.39	0.040	3.71	1.29	0.068

## References

[1] Chitre M. (2007). A high-frequency warm shallow water acoustic communications channel model and measurements. J. Acoust. Soc. Am..

[2] Stojanovic M., Preisig J. (2009). Underwater acoustic communication channels: Propagation models and statistical characterization. IEEE Commun. Mag..

[3] van Walree P.A. (2013). Propagation and scattering effects in inderwater acoustc communication channels. IEEE J. Ocean. Eng..

[4] Chitre M., Potter J., Ong S.H. Underwater acoustic channel characterisation for medium-range shallow water communications. Proceedings of the MTTS/IEEE Techno-Ocean’04.

[5] Yang W.B., Yang T.C. (2006). High-frequency channel characterization for M-ary frequency-shift-keying underwater acoustic communications. J. Acoust. Soc. Am..

[6] Hicheri R., Pätzol M., Talha B., Youssef N. A study on the distribution of the envelope and the capacity of underwater acoustic channels. Proceedings of the 14th IEEE International Conference on Communication Systems.

[7] Kim S.M., Byun S.H., Kim S.G., Kim D.J., Kim S., Lim Y.K. Underwater acoustic channel characterization at 6 kHz and 12 kHz in a shallow water near Jeju Island. Proceedings of the MTS/IEEE Oceans Conference.

[8] Kulhandjian H., Melodia T. Modeling underwater acoustic channels in short-range shallow water enviroments. Proceedings of the International Conference on Underwater Networks and Systems.

[9] Qarabaqi P., Stojanovic M. (2013). Statistical characterization and computationally efficient modeling of a class of underwater acoustic communication channels. IEEE J. Ocean. Eng..

[10] Kim J., Koh I.S., Lee Y. (2015). Short-term fading model for signals reflected by ocean surfaces in underwater acoustic communications. IET Commun..

[11] Hajenko T.J., Benson C.R. The high frequency underwater acoustic channel. Proceedings of the IEEE Oceans Conference.

[12] Ainslie M.A., Dahl P.H., de Jong C.A.F., Laws R.M. Practical spreading laws: The snakes and ladders of shallow water acoustics. Proceedings of the 2nd International Conference and Exhibition on Underwater Acoustics.

[13] Rappaport T.S. (1996). Wireless Communications: Principles and Practice.

[14] Goldsmith A. (2005). Wireless Communications.

[15] Simon M.K., Alouini M.S. (2004). Digital Communication over Fading Channels.

[16] Borowski B. Characterization of a very shallow water acoustic communication channel. Proceedings of the MTS/IEEE Oceans Conference.

[17] Yacoub M.D. (2007). The *κ*-*μ* and the *η*-*μ* distribution. IEEE Antennas Propag. Mag..

[18] Zhang J., Cross J., Zheng Y.R. Statistical channel modeling of wireless shallow water acoustic communications for experimental data. Proceedings of the IEEE Military Communication Conference.

[19] Ruiz-Vega F., Clemente M.C., Paris J.F., Otero P. Rician shadowed statistical characterization of shallow water acoustic channels for wireless communications. Proceedings of the UComms Conference.

[20] Paris J.F. (2014). Statistical characterization of *κ*-*μ* shadowed fading channels. IEEE Trans.Veh. Technol..

[21] Sánchez A., Robles E., Rodrigo F.J., Ruiz-Vega F., Fernández-Plazaola U., Paris J.F. Measurement and modeling of fading in ultrasonic underwater channels. Proceedings of the 2nd International Conference and Exhibition on Underwater Acoustic.

[22] Gradshteyn I., Ryzhik I.M. (2007). Tables of Integrals, Series and Products.

[23] Corder G.W., Foreman D.I. (2009). Nonparametric Statistics for Non-Statisticians: A Step-by-Step Approach.

[24] García-Corrales C., Cañete F.J., Paris J.F. (2014). Capacity of *κ*-*μ* Shadowed Fading Channels. Int. J. Antennas Propag..

